# Transition of Femtosecond-Filament-Solid Interactions from Single to Multiple Filament Regime

**DOI:** 10.1038/s41598-017-13188-4

**Published:** 2017-10-06

**Authors:** P. J. Skrodzki, M. Burger, I. Jovanovic

**Affiliations:** 1 Department of Nuclear Engineering and Radiological Sciences, University of Michigan, Ann Arbor, MI 48109 USA; 2 Center for Ultrafast Optical Science, University of Michigan, Ann Arbor, MI 48109 USA

## Abstract

High-peak-power fs-laser filaments offer unique characteristics attractive to remote sensing via techniques such as remote laser-induced breakdown spectroscopy (R-LIBS). The dynamics of several ablation mechanisms following the interaction between a filament and a solid determines the emission strength and reproducibility of target plasma, which is of relevance for R-LIBS applications. We investigate the space- and time-resolved dynamics of ionic and atomic emission from copper as well as the surrounding atmosphere in order to understand limitations of fs-filament-ablation for standoff energy delivery. Furthermore, we probe the shock front produced from filament-target interaction using time-resolved shadowgraphy and infer laser-material coupling efficiencies for both single and multiple filament regimes through analysis of shock expansion with the Sedov model for point detonation. The results provide insight into plasma structure for the range of peak powers up to 30 times the critical power for filamentation *P*_*cr*_. Despite the stochastic nucleation of multiple filaments at peak-powers greater than 16 *P*_*cr*_, emission of ionic and neutral species increases with pump beam intensity, and short-lived nitrogen emission originating from the ambient is consistently observed. Ultimately, results suggest favorable scaling of emission intensity from target species on the laser pump energy, furthering the prospects for use of filament-solid interactions for remote sensing.

## Introduction

High-peak-power fs-laser pulses propagated in air are of significant interest for many applications such as remote sensing^1,2^, remote laser-induced breakdown spectroscopy (R-LIBS)^3^, artificial lighting^4^, and others^5,6^. When such laser pulses propagate over extended distances in air, they are known to undergo the filamentation process^7^, which involves self-focusing and temporal compression due to the Kerr effect which also induces positive group velocity dispersion (GVD)^8^. The laser pulse propagates leaving a plasma column in its wake, referred to as the *filament*, which forms via mechanisms that include multiphoton ionization (MPI), electron avalanche ionization, and inverse bremsstrahlung^9,10^. Electron densities on the order of 10^15^–10^17^ cm^−3^ in the plasma introduce a negative contribution to GVD, stretching, and defocusing of the pulse^7,10^. The interplay of focusing and defocusing causes an oscillatory pattern of plasma generation at the onset of the filamentation process described by^8^

1 $${n}_{2}I=\frac{{\omega }_{p}^{2}}{2{\omega }^{2}}+\frac{1.22{\lambda }_{0}^{2}}{8\pi {n}_{0}{\omega }_{0}^{2}},$$

where the left-hand-side term corresponds to self-focusing due to the Kerr effect, while the two right-hand-side terms correspond to defocusing due to propagation through plasma and diffraction, respectively. Filamentation is a threshold phenomenon, since self-focusing requires a critical peak-power ${P}_{cr}={\lambda }_{0}^{2}\mathrm{(2}\pi {n}_{2})$, where *λ*_0_ is the pulse wavelength and *n*_2_ is the Kerr index of refraction^8,9^. The relevant critical power is predicted to be *P*_*cr*_ ∼ 3 GW at a central wavelength *λ*_0_ = 790 nm and with ${n}_{2}\,\sim \,3\times {10}^{-19}$ cm^2^ W^−1^ in air at standard temperature and pressure (STP)^9^.

The pioneering studies by Stelmaszczyk *et al*.^11^ and Rohwetter *et al*.^12,13^ demonstrated the capabilities of remote fs-filament ablation by observing atomic emission lines of copper and steel at a distance of 90 m. Compared to traditional ns-LIBS, detection of trace elements could also be achieved within similar (ppm) sensitivity^14^. Additionally, the robustness of filamentation-induced LIBS was demonstrated in simulated polar environment^15^. Recent interest in R-LIBS with solid targets for measurements in air^16–21^ highlights the need for fundamental understanding of the interaction between high-peak-power fs pulses exhibiting filamentation and solids. Noteworthy challenges in R-LIBS arise from intensity clamping in the filament core limited by MPI of air molecules^9^, instabilities in directed energy delivery for greater peak powers which lead to stochastic formation of multiple filaments^9,22^, and emission from atmospheric species from the filament and target plasmas^2,3,9,14^, which contributes to background in optical emission spectroscopy. The intensity-clamped filament core (*I* ∼ 10^13^ − 10^14^ W cm^−2^) is surrounded by an energy reservoir^23,24^, which refills core channels obstructed or perturbed by obstacles such as atmospheric water droplets^25^. The reservoir, which has a lower intensity than the core, often fails to overcome the ablation threshold of solid targets and contributes less toward ablation and may instead only heat the target surface^26^. This presents a challenge for R-LIBS since a part of the energy from the pulse from the surrounding reservoir may not contribute toward plasma formation. Another challenge in obtaining reproducible spectra for R-LIBS arises from the formation of multiple filaments, which cause non-uniform ablation. Multiple filamentation occurs when the peak power greatly exceeds 10^1^–10^2^ *P*_*cr*_; in this regime, several core channels form, each with increased power ${P}_{fil}\,\sim \,{\pi }^{2}{P}_{cr}\mathrm{/4}$ ^27^. Beam irregularities or perturbations nucleate these cores^22^.

In the previous work, Zhang *et al*. probed material ejection at early times (<10 ns) following fs-ablation^28^. However, typical timescales of interest for R-LIBS and other remote sensing applications involving filamentation are in the range of 100 ns–10 μs. Moreover, filamentation introduces additional complexity in ablation mechanisms resulting from a pattern of self-focusing and defocusing along the propagation distance as well as oscillation of the pulse duration. Mirza *et al*.^29^ conducted a comprehensive study of fs-ablation mechanisms for dielectric targets, but the concurrent literature lacks mature understanding of ablation with fs-filaments. Valenzuela *et al*.^26^ provided a comparison of ablation mechanisms for various focusing configurations of ultrashort pulses including slow or loosely focused as well as freely propagating filaments. Weidman *et al*.^18^ studied the crater formed following fs-filament ablation of GaAs with both loosely focused filaments (12 m standoff) and freely propagating filaments (50 m standoff). Xu *et al*.^30^ investigated the target plasma excitation temperature and electron density by studying the emission features of lead following loosely focused fs-filament ablation. Damage and breakdown thresholds of the target vary significantly with pump laser intensity as well as pulse duration. Cold ablation mechanisms (*i* .*e* . Coulomb explosion^31^ and fast atomization) dominate where filamentation is typically not present for pulse durations ≤10 ps^29^. To address these gaps in understanding, we explore the expansion of fs-filament-produced plasma from a copper target and correspondent shock over the timescales relevant to R-LIBS using two techniques: filtered spectral imaging and shadowgraphy. Ablation laser peak-powers span 20–100 GW (6–30 *P*_*cr*_), allowing the observation of the onset to steady formation of multiple filaments following loose focusing (*f*/100) in air. We demonstrate the effects of aforementioned challenges, including intensity clamping and multiple filamentation, on plasma expansion and uniformity as they pertain to application in R-LIBS and ultimately show that the emission from copper in air scales favorably with the increase in pulse energy as the transition from the single to multiple filament regime occurs.

## Results

### Shadowgraphy of filament-solid interaction

Two important parameters characterizing the R-LIBS performance are the reproducibility and the total emission intensity of the filament-produced plasma. We track the expansion of the shock front produced from the fs-filament-solid interaction near the copper target between 100 ns and 1 μs using a second-harmonic Nd:YAG probe laser pulse in order to observe the reproducibility of filament ablation. The probe incident on a CMOS detector reveals significant changes in the refractive index associated with sharp gradients of the air density, pressure, and temperature surrounding the target plasma. Supplementary Note 1 contains representative shadowgrams for the single and multiple filament regimes. We observe the formation and expansion of a secondary, internal shockwave from filament-solid interaction. Similar observations of internal shockwaves were made for ns-ablation of copper by Liu *et al*.^32^ and attributed to reflection from the back surface of the solid sample. Further complexity such as the formation and mixing of two distinct shockwaves is observed in the case of multiple filament formation for peak-powers exceeding 60 GW.

**Figure 1:**
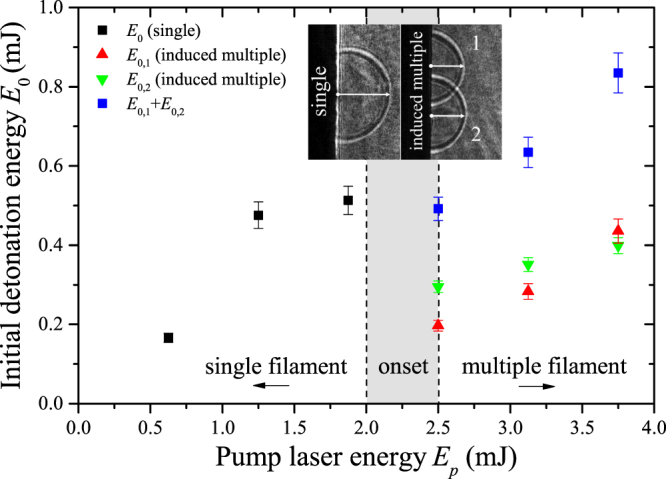
Initial detonation energy obtained from the predictive Sedov model. Focusing using an ordinary lens, resulting in random filament distribution in the multiple filament regime, is compared to focusing using a split lens, allowing induced multiple filament formation.

Furthermore, we quantify the laser-material coupling efficiency tracking the time-dependent expansion of the shock front. Laser-material coupling efficiency and the amount of ablated mass govern the second parameter of interest for R-LIBS, plasma emission. We assume spherical expansion from a point detonation in order to use the Sedov model (Supplementary Note 1) to estimate the energy deposited in the target. Notably, the assumed *t* ^0.4^ dependence may be a source of error in calculation of detonation energies. Nevertheless, the assumption proves reasonable for calculation of the detonation energy with *R* ^2^ > 0.97 in all cases. Figure 1 shows the measured initial detonation energy (subscripted 0) for each laser energy (subscripted *p*). The efficiency of laser-material coupling reaches ∼30 ± 5% in the single filament regime, where the energy loss in the filament is not considered. Several previous studies^33–36^ presented the limitations of the spherical approximation expansion beyond 100 ns. Chen *et al*.^35^ predicted two consecutive energy deposition steps for ns-ablation of water: surface evaporation dominant up to ∼200 ns, followed by phase explosion up to ∼1 μs. Their study compares laser energy deposited in each detonation stage, early detonation (*t* ≤ 200 ns) near the surface *E*_*s*_ and late (*t* > 200 ns) volume detonation *E*_*v*_, by determining the ratio of slopes of a linear Sedov model between early- and late-time experimental data *s* = (1+*E*_*v*_/*E*_*s*_)^0.2^ where *E*_0_ = *E*_*v*_ + *Es*. Upon similar analysis for energies in the single filament regime, we determine only a small change in the slope after 300 ns (∼0.8–7%) with increasing energy. This result implies single-stage detonation, which is consistent with cold ablation phenomena associated with fs-ablation. Plausible major mechanisms for efficiency loss include reflection from the sample surface as well as the weak contribution of the energy reservoir toward ablation. Copper has a high reflection coefficient ($\Re \,\sim \,$0.96) at 790 nm, dropping significantly with increased temperature^37–39^ as a result of the increase in electron-phonon collision rate.

**Figure 2:**
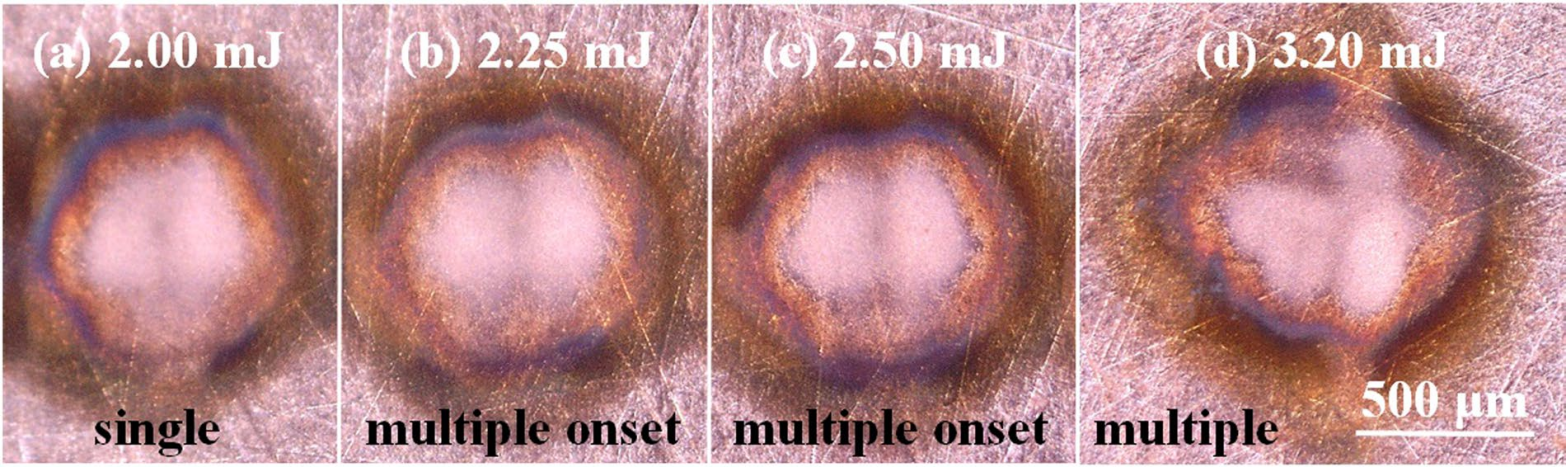
Microscope images of damage induced by fs-filament ablation. Images show damage induced by (**a**) a single filament (*E*_*p*_ = 2.00 mJ), (**b**)–(**c**) two filaments at the onset of multiple filamentation (*E*_*p*_ = 2.25 and 2.50 mJ, respectively), and (**d**) multiple filaments (*E*_*p*_ = 3.20 mJ).

The calculation of laser-material coupling efficiency does not account for energy loss to ionization and excitation in the filament. Here we estimate this energy loss to be equal to the energy needed to produce a plasma column with dimensions comparable to the filament observed in the experiment. We assume the mean energy needed to produce ionization (W-value) in dry air of 33.97 ± 0.05 J C^−1^ ^40^, filament core diameter of 100 μm^9^, a limiting electron density of 10^17^ cm^−3^ ^7,10^, and filament length of 5 cm, which was typically observed for 1.9 mJ pump laser energy. Furthermore, we assume the majority of ionization occurs locally, within the filament core. The resultant energy expended to ionization and excitation of this plasma column is ∼0.2 mJ, constituting ∼10% of the incident pulse energy. In this analysis we also assume a steep energy density gradient between the filament core and reservoir, as discussed in detail by Liu *et al*.^41^ and Luo *et al*.^42^. Liu *et al*. find that for the maintenance of the full length of the filament core which actually contains only ∼10% of the pulse energy, the propagation of a wide reservoir (whose transverse area is about 5–10 times as large as the filament core), in which up to 50% of the pulse energy is located together with the core, is necessary. Figure 2 (a) shows the damage from the various components of the single filament which are consistent with the filament dimensions predicted by Liu *et al*., a wider $\sim$300 μm-radius affected region from the core and reservoir. The focused irradiance on the copper surface is estimated to $\sim 5\times {10}^{13}$ W cm^−2^, assuming 10% of the remaining energy of the input pulse lies within the filament core of diameter 100 μm. This result compares well with the expected clamped intensity reported in several other works^23,24,26^.

In summary, ∼10% of the 1.9 mJ input energy is lost to ionization and excitation in the filament core, and the remaining energy either interacts with the target through absorption or reflection or is lost via diffraction from the outer edge of the filament. The Sedov calculation predicts ∼25–30% of the input energy contributes primarily to fast detonation in the single filament regime, leaving 60–65% of the energy lost to diffraction from the outer edge of the filament reservoir or reflection from the target surface. Modeling studies by Bogaerts *et al*.^43^ of ns-ablation of copper and by Cheng *et al*.^44^ of fs-ablation of copper predict a rapid drop in reflectivity of copper throughout ablation from ∼0.9 to 0.1 and from 0.85 to 0.23, respectively, due to a sharp temperature increase and surface roughening dependent on the incident laser fluence. Consequently, we cannot isolate the losses due to reflection and diffraction due to the dynamic absorption and reflection processes throughout ablation, but we conclude that the majority of the input energy does not contribute toward detonation.

**Figure 3:**
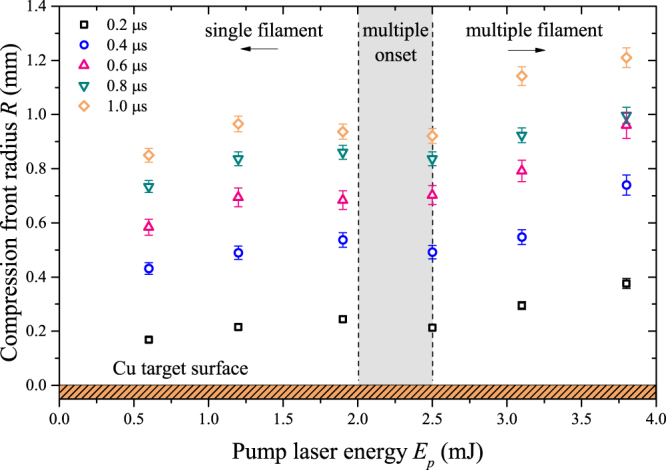
Dependence of the compression front radius on pulse energy. Several time steps (0.2–1.0 μs) are shown. The shaded region shows the energies in which the onset of multiple filamentation is observed. The figure emphasizes the deviation from the Sedov model in the random multiple filament regime.

With pulse energies 2.5 mJ or greater, random formation of multiple filaments is observed. Figure 2 shows damage on the sample from energies in the transition region from single to multiple filamentation. The irregular shape of the shockwave in the multiple filament regime violates the major assumption of shockwave symmetry in the point detonation approximation in the Sedov model. Instabilities and noise in the beam profile nucleate several plasma channels along the propagation direction of the beam, causing irregular target ablation as observed in Fig. 2(d). Fig. 3 emphasizes the deviation from the Sedov model for point explosions by comparing the compression front radii measured similarly between the single and multiple filament regimes from the target to the shock position along the laser axis as shown by the inset in Fig. 1. Evidently, it is no longer appropriate to calculate the detonation energies under the assumption of symmetric shock expansion from a point because of the irregular shape. In order to maintain the spherical expansion model in the multiple filament regime, we consistently generate two sufficiently separated shock fronts with a beam focused by a split lens, effectively inducing multiple filamentation. The details of this experimental approach are presented in Supplementary Note 1. The two distinct shock fronts are each seeded by individual filament cores, and the spherical expansion model is used for each. Subsequently, the individual and total detonation energies are presented in Fig. 1 for pump laser energies comparable to those in which we observed random multiple filamentation.

### Spectral imaging of plasma formed from filament-solid interaction

**Figure 4:**
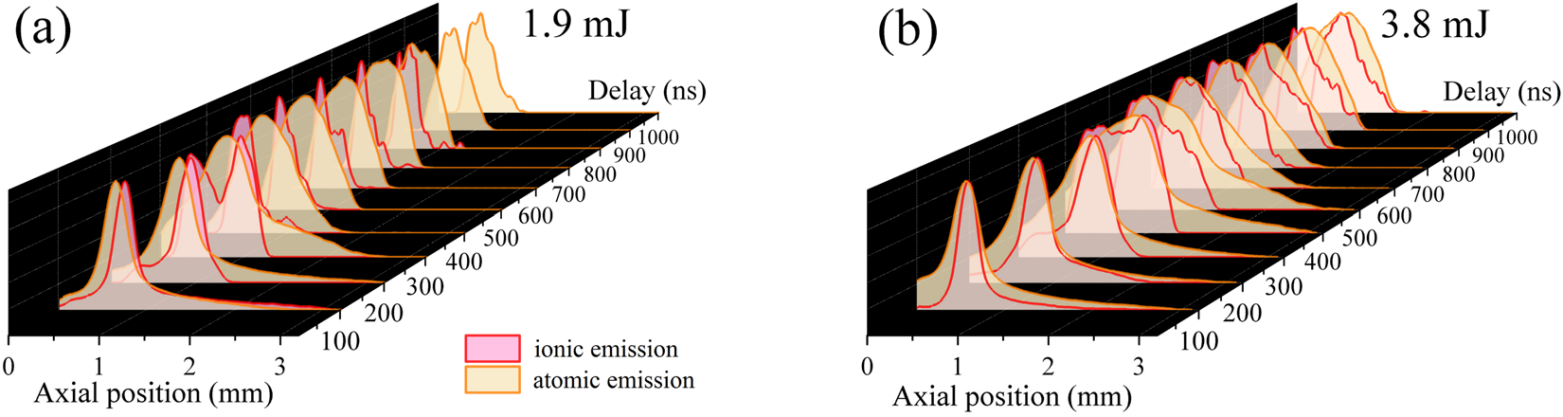
Normalized central axis emission from target species. Time-resolved, normalized emission distributions along the central axis of each plume for (red) ionic and (orange) neutral species at pulse energies of (**a**) 1.9 mJ and (**b**) 3.8 mJ representative of the single and multiple filament regimes, respectively.

Next, we observe directly the species-distinct time- and space-resolved emission from the filament-produced plasma using filtered and gated spectral imaging. Supplementary Note 2 provides time-resolved frames of neutral, ionic, and atmospheric species in the target plasma. Figure 4 shows the normalized emission from ionic and neutral species from the target integrated along the central plane (perpendicular to the image plane) of the plume for 1.9 and 3.8 mJ laser pulse energies representative of the single and multiple filament regimes, respectively. Figure 4 emphasizes the spatial distribution of species as a function of time providing a macroscopic view of plasma components which constitute signal in applications such as R-LIBS. In both single and multiple filament regimes, emission from ions is localized near the plume front. Ionic emission quickly dissipates near the outer plume boundary, revealing an emission track along the central axis toward the target (prevalent in Fig. S5 of Supplementary Note 2). This track suggests preferential ionization in the wake of the filament channel. Neutral emission at earlier delays is also localized and trails the maximum of ionic emission.

**Figure 5:**
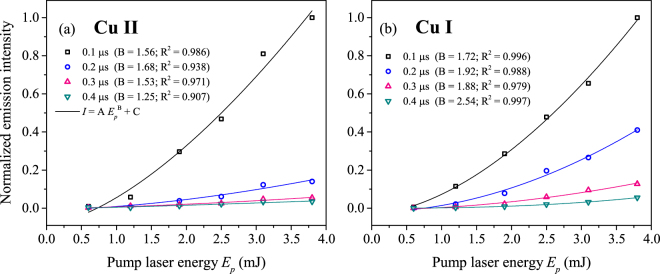
Pulse energy dependence of target species’ emission intensity. Total emission from (**a**) ionic (Cu II) and (**b**) neutral (Cu I) target species as a function of pump laser energy.

Furthermore, we compare the total emission of each species to pump laser energy in Fig. 5 in order to understand how laser-material coupling affects signal considering R-LIBS applications. Despite the apparent bifurcation of species and plasma non-uniformity in the case of multiple filamentation (Fig. 4), the total emission of each species exhibits no abrupt transition from single to multiple filament regime, as seen in Fig. 5. The continuous increase in emission intensity in the single filament regime shows that intensity clamping negligibly affects plasma formation. Moreover, the power-law trend of total emission with respect to laser pulse energy observed in the multiple filament regime is in agreement with the trend of the initial detonation energies calculated from controlled formation of multiple filaments with respect to laser pulses energy (Fig. 1). This implies that, while the energy is divided into multiple filament cores, the contribution of individual cores to the overall emission is additive in nature.

## Discussion

The results prove interesting toward the understanding of plasma formation and dynamics with loosely focused filaments, with important implications on the use of filaments for R-LIBS. The single filament regime yields reproducible, symmetric plasma about the central axis confined within the shock front. The shock front expands according to the Sedov model for point detonation, approaching asymptotically the speed of sound in the the medium (*c* = 343 m s^−1^ at STP). The pressure behind the shock front increases asymptotically to match the initial pressure, and the shock ultimately degenerates into a spherical acoustic wave. Analysis of the rate of expansion of the shock in the single filament regime predicts laser-material coupling efficiency of ∼25–30% which suggests ∼15–20% contribution from the energy reservoir assuming the filament core contains ∼10% of the input pulse energy. Dominant mechanisms for energy loss include ionization and excitation of air to form the filament plasma, diffraction from the outer edge of the filament as discussed by Liu *et al*.^41^, and reflection from the target surface.

The emission from the target favors neutral target species; similar observations were made in the past by Harilal *et al*.^21^ and Stelmaszczyk *et al*.^11^. Ions are ejected from the target and disappear quickly, revealing an emission track toward the target along the filament channel, suggesting preferential ionization in the wake of the filament which perturbs the electric field in the medium along its path. The degree of ionization at the plume front drops rapidly, resulting in notable redistribution of both ionic and atomic emission. This bifurcation is apparent in the normalized emission intensity distribution in Fig. 4. We estimate the axial velocities of both emission species (position of maximum of emission intensity for each) and the compression front in the 100–300 ns time interval after the laser pulse. The velocities of atomic and ionic species’ emission peaks range from 5 to almost 7 Ma, respectively. On the other hand, the shock front velocities do not exceed 6.5 Ma even in the case of the highest pump laser energy (3.8 mJ). Consequently, the deceleration of the shock front allows the ablated species to catch up, interact with the boundary layer, and eventually scatter, de-excite or recombine. This phenomenon may justify the rapid decline in ionic emission intensity at the plume-shock border. Similar observations are reported for ns-ablation and attributed to a high-temperature region shift towards the target surface^45^. Moreover, this species bifurcation and large contribution of early-time surface detonation suggest the dominance of fast ablation mechanisms. However, the formation of internal shock fronts, the small contribution of late volume detonation, and the ionic emission track in the wake of the filament channel suggest the occurrence of several ablation mechanisms well-separated in time as observed for ns-ablation of copper by Liu *et al*.^32^. Arnold *et al*.^46^ provide an analytical review of spherical expansion of a vapor plume and corresponding shock outlining mechanisms for formation of internal shockwaves, namely reflection from the back surface of a sample with finite thickness. Typical cold ablation mechanisms for pulse durations ≤10 ps include Coulomb explosion and immediate atomization, whereas ablation mechanisms for longer pulse durations involve lattice heating and consequent phase explosion^29^. Coulomb explosion and atomization cause early ejection of ions from the target, explaining the significant ion density at the front of the plume. Later ablation mechanisms related to lattice heating occur following the interaction of the filament plasma with the target; also, interaction between the filament and target plasmas plausibly explains the ionic emission track in the wake of the filament channel.

Typical long-distance remote measurements require peak-powers well above the threshold for multiple filamentation^9,22,47^. The multiple filament regime (reached at pulse energies ≥2.5 mJ in this study) exhibits a more complex mixing of several shock fronts from distinct filament channels no longer accurately represented by the Sedov point explosion model. However, the total energy calculated from controlled multiple filamentation as a function of initial pulse energy exhibits a positive trend that correlates with the total emission intensity of ionic and neutral target species in Fig. 5(a) and (b), respectively. This comparison reveals that the saturation in intensity for this range of energies limits only the energy loss to excitation and ionization of air along the filament and has no detrimental effects toward target ablation for application in R-LIBS. Despite the sporadic formation of multiple filaments in this regime, the emission intensity from both species exhibits no associated aberrant behavior and scales favorably with measured initial detonation energy. Successive effort toward understanding fundamental plasma properties at early times following initial filament-target interaction is required. Measuring plasma temperature and electron density in the earliest (sub-ns) stages may reveal initial conditions governing the expansion of the shock front and plume observed in this work. Although this study observes the interaction between a filament produced under specific focusing conditions and a copper target, the results yield understanding which may have more general importance to explaining filament ablation mechanisms.

## Methods

### Filament production and target ablation

We employed the custom-built laser system Lambda Cubed at the Center for Ultrafast Optical Science at the University of Michigan (Ann Arbor, Michigan, USA). The system incorporates two stages of Ti:sapphire-based chirped-pulse amplification including a primary regenerative amplifier and secondary three-pass amplifier, capable of delivering pulses up to 20 mJ pulses at 0.5 kHz repetition rate. The operational conditions in our experiment are as follows: pulse energies in the range of 0.6–3.8 mJ, 40 fs (FWHM) pulse duration, central wavelength of 790 nm, 80 Hz repetition rate, and linearly-polarized beam.

**Figure 6:**
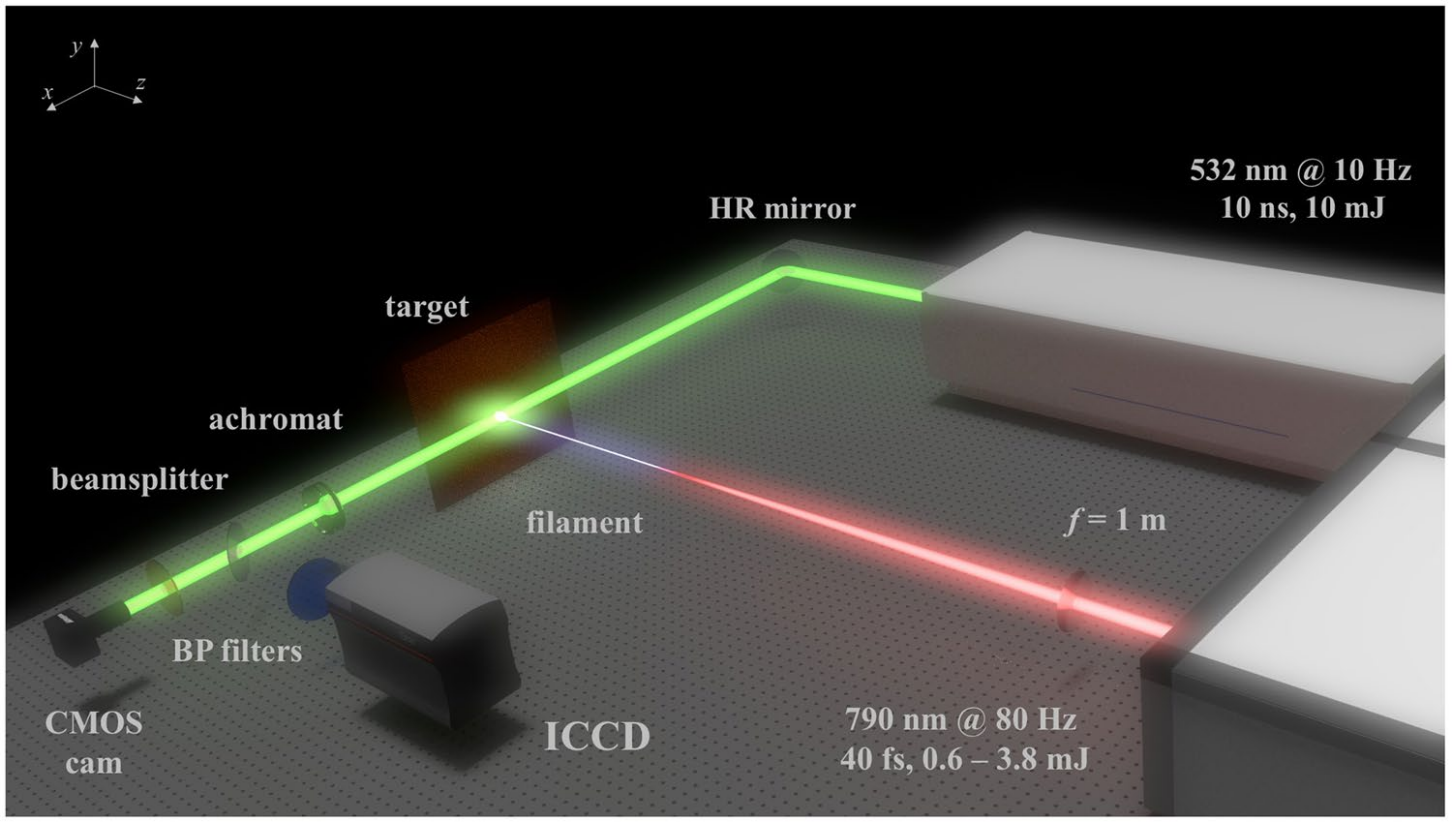
Schematic of the experimental setup. The dual-imaging system employs a probe laser and CMOS detector for shadowgraphy, tracking the shock front produced from filament-target interaction, and ICCD for viewing emission from isolated target species in the corresponding target plasma. Species are isolated by selective bandpass (BP) filters placed in front of the ICCD. High-reflectivity (HR) mirrors allow for alignment of the probe beam used in shadowgraphy.

Filaments were formed in air by focusing the laser beam with a 1 m focal length lens (*f*/100). A pure polished copper sample was placed at the geometrical focus of the lens. The focused filament impinging the sample surface formed the plasma. Figure 6 shows a schematic of the one-to-one plasma imaging system employing a 7.5 cm achromatic lens. The setup enables taking both shadowgraphic and plasma emission recordings either simultaneously (via beamsplitter), or separately (via flippable mirror). An x-y-z translation stage allowed for continuous target movement during the measurements.

### Shadowgraphic imaging

Shadowgraphy used a second-harmonic probe beam from an Nd:YAG laser (Spectra Physics Quanta-Ray, 10 mJ, 10 ns pulse duration, 10 Hz repetition rate). The 8-mm diameter probe beam was incident onto a 1280 × 960 pixel CMOS detector (Mightex CGE-B013-U, 3.75 m pixel width). A laser-line filter was coupled to the detector in order to attenuate plasma emission. The probe was synchronized to every eighth pulse from the 80-Hz Lambda Cubed laser system. Images track the evolution of the shock front formed by the filament-target interaction at 100-ns increments following the onset of plasma formation up to 1 μs.

### Spectral imaging

Plasma spectral emission was imaged side-on onto an ICCD (Andor iStar 334 T, 1064 × 1064 pixels, 13 m pixel width), cooled to −25 °C. Spectroscopic images of different emitting species were obtained using narrow-band optical filters placed in front of the ICCD^48^. The spectral lines selected as representative of the emission of the neutral species include Cu I 521.82 nm (4*p* ^2^ ${{\rm{P}}}_{\mathrm{3/2}}^{0}$ – 4*d* ^2^ D_5/2_) and N I 744.23 nm (3*s* ^4^ P_3/2_ – 3*p* ^4^ ${{\rm{S}}}_{\mathrm{5/2}}^{0}$). Behavior of ions is monitored by observing Cu II 490.97 nm (4*f* ^3^ H_6_ – 4*d* ^3^ G_5_) line emission. Central wavelengths of the narrow band filters are 520 nm, 490 nm, and 750 nm for Cu I, Cu II and N I, respectively. Gate width for each step was 3 ns, and 30 accumulations constitute a single image. Every image was normalized to its own maximum, and each species is represented by a different false color scheme. Adequate gain level is determined experimentally and maintained constant among measurement steps of 100 ns.

## Electronic supplementary material

 Supplementary Information
