# Supplementary material for: Transition of Femtosecond-Filament-Solid Interactions from Single to Multiple Filament Regime
Source: Sci Rep. 2017 Oct 6;7:12740. doi: 10.1038/s41598-017-13188-4 (PMC5630638; doi:10.1038/s41598-017-13188-4)
Supplement: Supplementary file 1 — Supplementary Information [file 41598_2017_13188_MOESM1_ESM.pdf]

Transition of Femtosecond-Filament-Solid Interactions from Single to Multiple Filament Regime

Supplementary Information

P. J. Skrodzki[1,2,*], M. Burger[1,2], and I. Jovanovic[1,2]

[1]Department of Nuclear Engineering and Radiological Sciences,
University of Michigan, Ann Arbor, MI 48019, USA
[2]Center for Ultrafast Optical Science,
University of Michigan, Ann Arbor, MI 48109, USA
[*]pskrodzk@umich.edu

Supplementary Note 1: Calculation of deposited energy and generation of reproducible multiple filamentation

Fig. S1 shows representative time-resolved shadowgraphic images in which the single and the multiple filament regimes can be distinguished. Those regimes correspond to pump laser energies of 1.9 and 3.8 mJ, respectively. The curves in Fig. S2 predict the time-dependent axial expansion of the shock front approximated as a point explosion with the Sedov model:

$$R = \xi \left(\frac{E_0}{\rho}\right)^{0.2} (t - t_0)^{0.4} + R_0, \tag{S1}$$

where R represents the shock front radius at delay t with initial explosion energy E_0 and medium density ρ, while ξ represents a dimensionless fitting parameter obtained from the adiabatic ratio γ for the medium in the initial conditions [S1]:

$$\xi \approx \left[\frac{75}{16\pi}\frac{(\gamma-1)(\gamma+1)^2}{3\gamma-1}\right]^{0.2}. \tag{S2}$$

The initial conditions we use for the Sedov model correspond to air near STP: density of 1.225 kg m^{-3} and adiabatic ratio of 1.40, yielding $\xi \approx 1.01$. Notably, shadowgraphy shows the expected positive and negative shock components corresponding to a compression wave followed by a rarefaction wave, as shown by the inset in Fig. S2 (b). The measured radii correspond to the distance between the target and the minimum intensity of the rarefaction front and the maximum intensity of the compression front along the laser axis. Data points represent the mean of three three measured radii at each time step and pump laser energy with error representing the first standard deviation from the mean. Although each measurement represents a different shot on a fresh target position, the fluctuation in the shock front position remains below 7% despite the sporadic asymmetry of the shock profile in the multiple filament regime. The aberrant behavior is more prevalent in the deviation from the Sedov model because the point detonation approximation is no longer accurate in this multiple filament regime: In the extreme case, multiple filament cores are sufficiently separated to produce distinct shock waves as shown in Fig. S3 (a). We exclude these

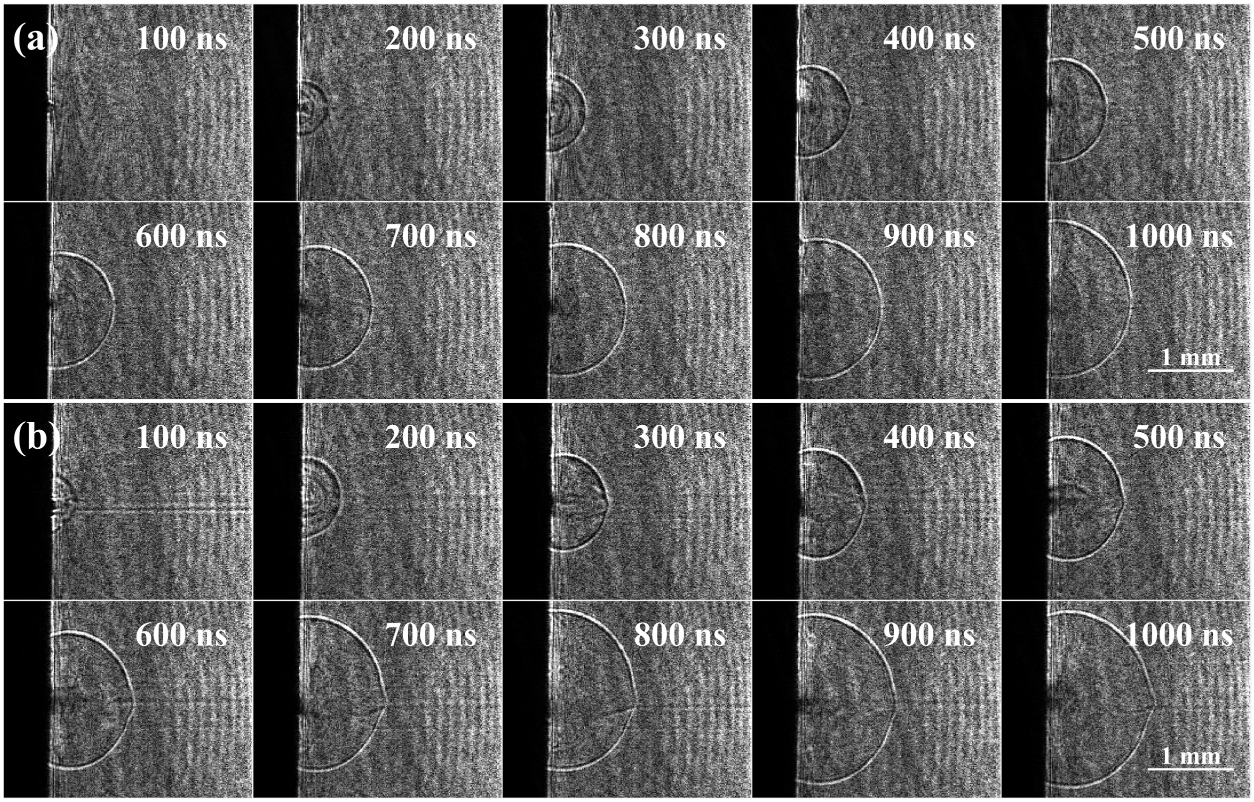

Figure S1: **Shadowgraphy of filament-solid interaction.** Frames depict spherical shock front expansion following interaction of filament formed by (a) 1.9 mJ (single-filament regime) and (b) 3.8 mJ (multiple-filament regime) pulse energies with a copper target.

frames from our Sedov analysis and instead replicate the conditions for formation of two spatially-resolved shocks discussed later. Although the shock radius alone does not fully characterize the asymmetry of the shock, the asymmetry in the case of multiple filamentation is manifested in Fig. S2 as more rapid expansion than predicted by the Sedov model. The shock radius is measured along the laser axis from the target to the shock position similarly between single and multiple filament regimes shown by the inset of Fig. 1 of the main text. Fig. S1 (b) better shows this asymmetry, although only qualitatively. The energy deposited in the target (presented in the main text) is calculated for compression front radii.

In order to better maintain shot-to-shot reproducibility in the multiple filament regime and be able to apply the assumption of spherical expansion, we generate consistent multiple filament patterns by deliberately seeding the nonuniformities in the beam. Focusing via a split lens ($f\# \sim 100$) yields consistent formation of sufficiently separated filament channels, as shown in Fig. S4. Each filament produces a separate shock wave; the centroids of the two shock waves are separated $\sim$0.6 mm vertically and $\sim$0.6 mm horizontally (into the page), so that the interaction between shock fronts may be neglected, as shown in Fig. S3 (b). Analysis of the individual compression front radii from these experiments shows increasing initial detonation energy in the multiple filament regime with input energy. Ultimately, we compare the energy deposited to total emission intensity of each target species and observe a similar dependence between them, suggesting that although the energy is distributed into multiple filament channels, the total emission results from the simple summation of the individual contributions of those two channels. Notably, although we measure laser energy

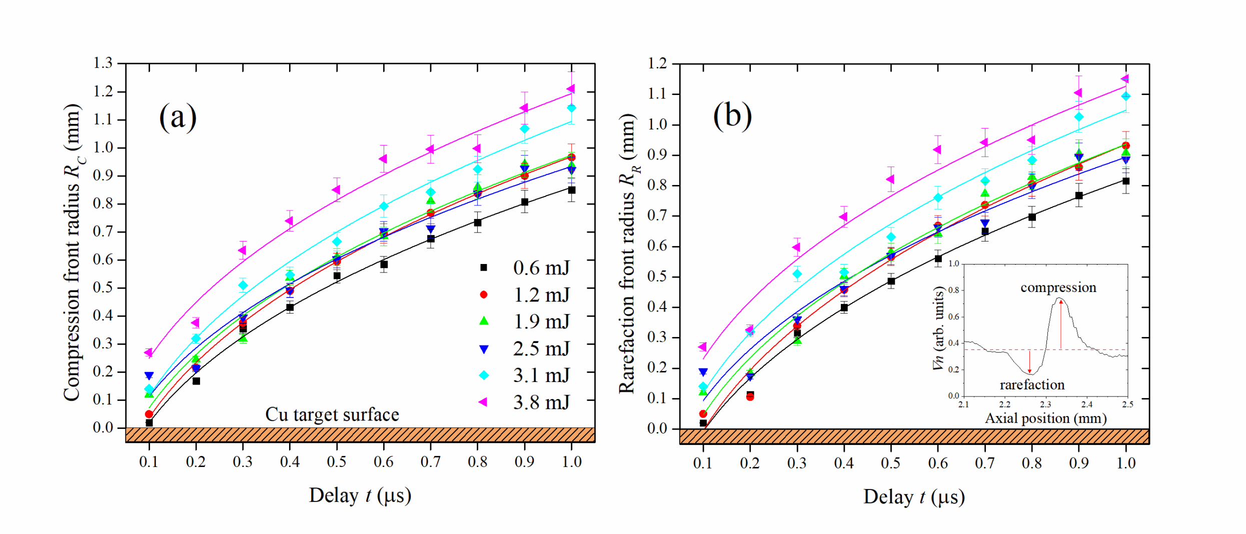

Figure S2: **Evolution of shock front radius.** (a) Compression and (b) rarefaction front radii for pulse energies ranging from 0.6–1.9 mJ in the single filament regime are in good agreement with the Sedov model; radii for pulse energies >1.9 mJ representative of the random multiple filament regime are no longer appropriately represented by the Sedov model motivating experiments with the split lens inducing multiple filamentation. Inset figure shows the gradient in refractive index at the shock front, depicting compression and rarefaction regions for pulse energy of 0.6 mJ and delay of 300 ns.

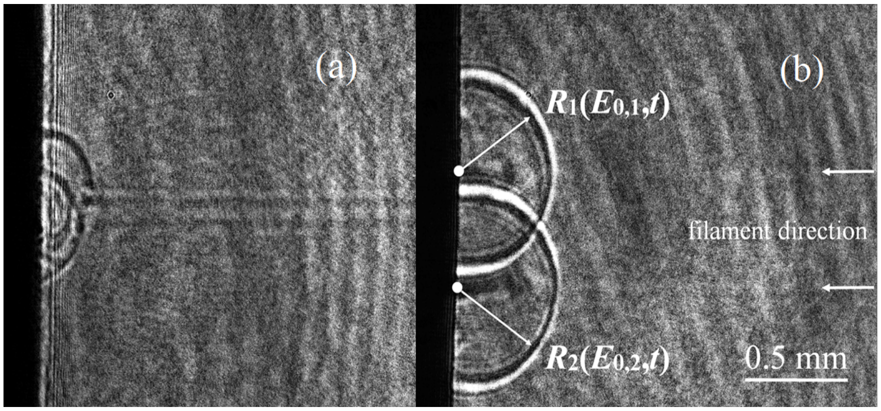

Figure S3: **Multiple filament channels producing two distinct shock waves.** (a) Random formation of two shock fronts from individual filament channels in the multiple filament energy regime (3.1 mJ) with probe and camera delay of 200 ns; (b) controlled formation of two distinct shock fronts from a split beam with a pulse energy of 3.1 mJ and recorded with 400 ns probe delay.

after the lens, the sharp boundary between the halves of the split lens causes diffraction which reduces the energy reaching the target by a small fraction for which we cannot fully account.

Supplementary Note 2: Time- and space-resolved characteristics of plasma emission

Fast spectral imaging performed up to 1 μs after the laser pulse (Fig. S5) reveals spatially-resolved emission of target species from the fs-filament-produced plasma. The time-dependent total emission

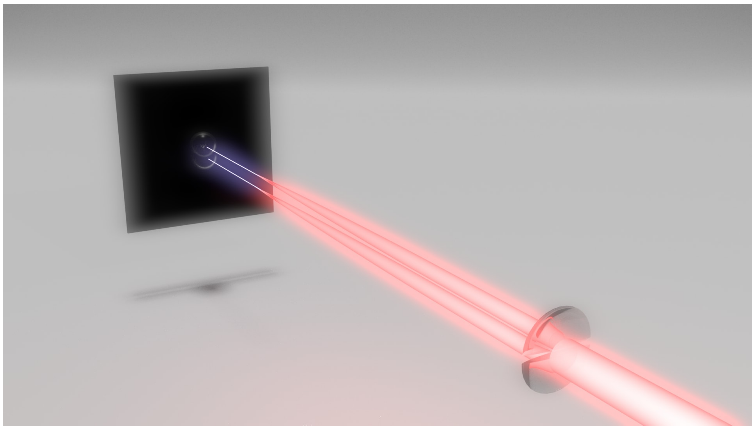

Figure S4: **Inducing two filaments using a split lens.** The input beam was focused via a split lens in order to consistently generate two filament cores, each yielding a distinct shock front for which we could assume the spherical expansion model following point detonation (Fig. S3). The input beam energies were similar to those in which we observed random multiple filamentation and measured after the split lens.

of both ionic (Cu II 490.97 nm) and atomic (Cu I 521.82 nm) species decays exponentially; however, ionic emission dissipates more quickly than neutral emission. Fig. S5 (a) shows the ionic (top) and neutral (bottom) emission representative of the single filament regime in which the plasma emission is relatively short-lived. Fig. S5 (b) shows the emission from both species before the onset of multiple filamentation. The plume following neutral emission expands from the target with the shock front before detaching beyond $\sim$400 ns and exhibits a spherical shape. Fig. S5 (c) shows emission in the multiple filament regime; emission from both species appears asymmetric during expansion, corresponding to the random seeding of several filament channels, as also observed via shadowgraphy.

Atomic emission of nitrogen (N I 744.23 nm) originating from the ambient air is observed at pulse energies beyond 1.9 mJ. Fig. S6 shows the short-lived emission from atmospheric nitrogen for two energies (1.9 and 3.8 mJ) representative of the single and multiple filament regimes, respectively.

Confined emission from nitrogen species from the ambient air indicates strong localization of kinetic energy within the plume of ablated species and a weak interaction (intermixing) boundary with the surrounding gas located at the plume front. The absence of plasma shielding [S2, S3] and laser induced absorption waves [S4] in the case of fs-ablation reduces the probability of intense collisional ionization of the background gas in the boundary region. Similar observations were reported in previous studies using either loose [S5] or tight focusing [S6] in air and argon environments, respectively. Moreover, the brevity of emission from the ambient bodes well for R-LIBS applications with solid targets, since the emission from the ambient can congest the spectra and overwhelm the emission from trace target species.

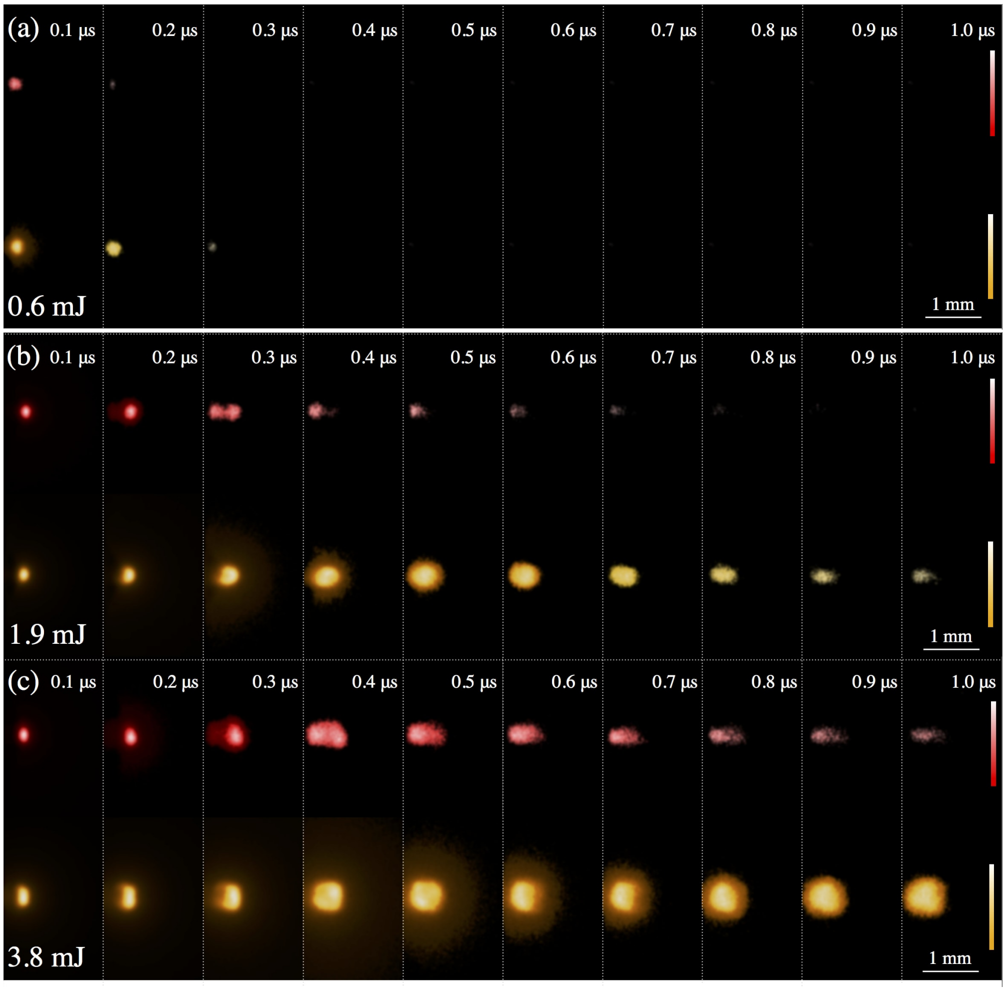

Figure S5: **Dynamics of spectral emission species from the target.** Emission from (top, red hue) ionic target species (Cu II 490.97 nm) and (bottom, orange hue) neutral target species (Cu I 521.82 nm) for initial pulse energies: (a) 0.6 mJ, (b) 1.9 mJ, and (c) 3.8 mJ.

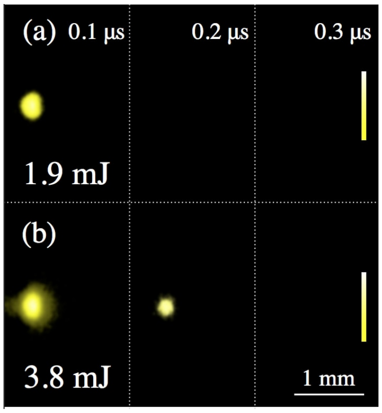

Figure S6: **Dynamics of spectral emission from the surrounding gas.** Emission from N I 744.23 nm representative of atmosphere excited by the filament-target interaction at pulse energies of (a) 1.9 mJ and (b) 3.8 mJ.

Supplementary References

[S1] L. I. Sedov. *Similarity and Dimensional Methods in Mechanics.* Academic Press, 1959.

[S2] G. Cristoforetti, G. Lorenzetti, P. A. Benedetti, E. Tognoni, S. Legnaioli, and V. Palleschi. Effect of laser parameters on plasma shielding in single and double pulse configurations during the ablation of an aluminium target. *Journal of Physics D: Applied Physics*, 42(22):225207, 2009. doi: 10.1088/0022-3727/42/22/225207.

[S3] M. Burger, D. Pantić, Z. Nikolić, and S. Djeniže. Role of spectroscopic diagnostics in studying nanosecond laser-plasma interaction. *The European Physical Journal D*, 2017, in press. doi: 10.1140/epjd/e2017-70750-5.

[S4] J.-F. Y. Gravel and D. Boudreau. Study by focused shadowgraphy of the effect of laser irradiance on laser-induced plasma formation and ablation rate in various gases. *Spectrochimica Acta Part B: Atomic Spectroscopy*, 64(1):56–66, 2009. doi: 10.1016/j.sab.2008.10.037.

[S5] K. Stelmaszczyk, P. Rohwetter, R. Ackermann, G. Mejean, J. Yu, E. Salmon, J. Kasparian, J.-P. Wolf, and L. Woste. Non-linear effects accompanying terawatt laser-pulse in air and their applications. *Proceedings SPIE 6158*, 6158(0F), 2004. doi: 10.1117/12.675772.

[S6] N. L. LaHaye, S. S. Harilal, P. K. Diwakar, and A. Hassanein. Characterization of laser ablation sample introduction plasma plumes in fs-la-icp-ms. *Journal of Analytic Atomic Spectrometry*, 29:2267–2274, 2014. doi: 10.1039/C4JA00200H.
